# Hypercatabolism and Anti-catabolic Therapies in the Persistent Inflammation, Immunosuppression, and Catabolism Syndrome

**DOI:** 10.3389/fnut.2022.941097

**Published:** 2022-07-13

**Authors:** Jinlin Zhang, Wenchen Luo, Changhong Miao, Jing Zhong

**Affiliations:** ^1^Department of Anesthesiology, Zhongshan Hospital Fudan University, Shanghai, China; ^2^Fudan Zhangjiang Institute, Shanghai, China; ^3^Department of Anesthesiology, Zhongshan Wusong Hospital, Fudan University, Shanghai, China; ^4^Shanghai Key Laboratory of Perioperative Stress and Protection, Shanghai, China

**Keywords:** chronic critical illness, persistent inflammation, immunosuppression, and catabolism syndrome, hypercatabolism, mitochondrial dysfunction, gut dysfunction, anti-catabolic therapy

## Abstract

Owing to the development of intensive care units, many patients survive their initial insults but progress to chronic critical illness (CCI). Patients with CCI are characterized by prolonged hospitalization, poor outcomes, and significant long-term mortality. Some of these patients get into a state of persistent low-grade inflammation, suppressed immunity, and ongoing catabolism, which was defined as persistent inflammation, immunosuppression, and catabolism syndrome (PICS) in 2012. Over the past few years, some progress has been made in the treatment of PICS. However, most of the existing studies are about the role of persistent inflammation and suppressed immunity in PICS. As one of the hallmarks of PICS, hypercatabolism has received little research attention. In this review, we explore the potential pathophysiological changes and molecular mechanisms of hypercatabolism and its role in PICS. In addition, we summarize current therapies for improving the hypercatabolic status and recommendations for patients with PICS.

## Introduction

With the improvement in intensive care unit (ICU) technology in the past few decades, many critically ill patients survive initial insults but develop chronic critical illness (CCI). These patients experience more complications, prolonged ICU stays (>14 days), ongoing organ dysfunction, and significant long-term mortality (1). It is estimated that 30–50% of patients with CCI are characterized by persistent low-grade inflammation, suppressed immunity, and ongoing catabolism despite nutritional interventions (2, 3). In 2012, Gentile et al. (4) postulated persistent inflammation, immunosuppression, and catabolism syndrome (PICS) to describe this subset of patients with CCI. PICS has received a lot of attention since it was proposed. PICS may be secondary to several acute events, such as severe blunt trauma, severe burns, severe acute pancreatitis, and sepsis in particular. Poor premorbid health conditions and an age of at least 65 years are considered to be clinical risk factors for PICS (5–7). Several mildly different diagnostic criteria for PICS have been reported and are summarized in Table 1.

**TABLE 1:** Diagnostic criteria for PICS.

	Gentile et al. (4)	Mira et al. (3)	Mira et al. (115)	Varela et al. (116)	Nakamura et al. (117)
ICU stay (day)	>14	>14	>14	≥10	>14
CRP (mg/dl)	>0.15	>0.05	>0.05	>0.15	>3.0
Total lymphocyte count (× 10^9^/L)	<0.8	<0.8		<0.8	<0.8
Serum albumin (g/dl)	<3.0	<3.0	<3.0	<3.0	<3.0
Pre-albumin (mg/dl)	<10	<10			<10
Creatinine height index	<80%	<80%	<80%	<80%	<80%
Retinal binding protein (mg/dl)	<0.01		<1	<0.01	<0.01
Weight loss	>10%	>10%	>10%	>10%	>10%
BMI during hospitalization	<18	<18	<18	<18	<18

*After unifying the units, there are some differences in ICU stay, C-reactive protein (CRP) levels, and retinal binding protein levels among these criteria.*

*PICS, persistent inflammation, immunosuppression, and catabolism syndrome; ICU, intensive care unit; CRP, C-reactive protein; BMI, body mass index.*

Persistent inflammation, immunosuppression, and catabolism syndrome has been plaguing ICU doctors and patients owing to its poor prognosis and associated treatment difficulty. Hence, it is essential to clarify the pathophysiology of PICS. To date, most of the existing literature focuses on persistent inflammation and suppressed immunity in PICS, and the role of hypercatabolism has not yet been reviewed. In this article, we reviewed the pathophysiological changes of hypercatabolism and their effects on persistent inflammation and immunosuppression in PICS, aiming to offer a mechanistic framework for PICS. Furthermore, we summarized current therapies aimed at improving hypercatabolic conditions in patients with PICS.

## Methods

Data were acquired from PubMed, MEDLINE, Scopus, and OVID using the following search terms: persistent inflammation, immunosuppression, and catabolism syndrome, (persistent inflammation, immunosuppression, and catabolism syndrome) AND (metabolism OR catabolism), (chronic critical illness OR sepsis) AND (metabolism OR catabolism), (persistent inflammation, immunosuppression, and catabolism syndrome OR chronic critical illness OR sepsis) AND (muscle wasting OR muscle atrophy OR muscle mass), (persistent inflammation, immunosuppression, and catabolism syndrome OR chronic critical illness) AND (therapy). There was no restriction on the type of article and the study design. Articles from all years were considered, especially those from the last decade.

## Hypercatabolism and Its Pathophysiological Changes in Persistent Inflammation, Immunosuppression, and Catabolism Syndrome

Critically ill patients in an overall hypercatabolic state show a significant decomposition of macronutrients, including protein stores, carbohydrates, and lipids (8, 9). Evident proteolysis, a major characteristic of PICS, is best documented in skeletal muscles (10). Patients with PICS often present with profound muscle wasting and weight loss during their hospitalization, despite the administration of enteral nutrition (11). Several mechanisms related to the hypercatabolic state in patients with PICS are summarized as follows:

### Inflammation Contributes to Skeletal Muscle Wasting

Both infectious and non-infectious insults induce a persistent inflammatory response in patients with PICS. Elevated inflammatory cytokine levels in the circulation have been shown to be associated with skeletal muscle wasting in critically ill patients (12) and also to induce muscle atrophy by activating pro-atrophy transcription factors, signal transducer and activator of transcription (STAT) and nuclear factor-kappa B (NF-κB) (13). Among these cytokines, tumor necrosis factor α (TNF-α), interleukin 1 (IL-1), and interleukin 6 (IL-6) are the most investigated. As an important signaling molecule, IL-6 can bind to the α-receptor and β-receptor glycoprotein 130 (gp130), after which it activates Janus kinase (JAK) and contributes to the phosphorylation of STAT (14). Under normal conditions, the IL-6 in skeletal muscle cells regulates skeletal muscle energy metabolism and promotes muscle growth and hypertrophy (15, 16). However, persistently elevated IL-6 levels will increase mitochondrial reactive oxygen species (ROS) production and oxidative stress in muscle cells (17). Recent studies have demonstrated that IL-6 induces skeletal muscle atrophy by activating the IL-6/gp130/JAK2/STAT3 pathway (17, 18).

Inflammatory cytokines also inhibit the mammalian target of the rapamycin (mTOR)-mediated signaling pathway to decrease protein synthesis. mTOR is a major regulator of protein synthesis in skeletal muscles (13). Amino acids, insulin, and insulin-like growth factor-1 (IGF-1) can activate mTOR and upregulate protein synthesis (9). Among these activators, the insulin and IGF-1-mediated mTOR signaling pathway is considered to be a key hub for protein synthesis and degradation (19). Whether or not muscle proteins are synthesized primarily depends on the activity of the IGF-1/phosphoinositide 3-kinase (PI3K)/Akt/mTOR signaling pathway (20). However, the activity of mTOR is suppressed by inflammatory cytokines, and this decreases protein synthesis (9). Eventually, elevated inflammatory cytokine levels in patients with PICS contribute to skeletal muscle wasting by increasing protein degradation and decreasing protein synthesis.

### Hormonal Changes Induce the Hypercatabolic Status

Many released pro-inflammatory cytokines cause changes in the activities of the endocrine and automatic nervous systems in patients with sepsis and septic shock, causing changes in the levels of hormones (21, 22). In the chronic stage of critical illness, the neuroendocrine system is generally inhibited, especially the hypothalamic-pituitary axis (HPA), resulting in decreased levels of the downstream corresponding hormones (23). Decreased levels of thyroid hormones, growth hormones, and sex hormones were observed in patients with CCI (23). These three categories of hormones are known to play important roles in metabolism and skeletal muscle growth. Low thyroid hormone levels were found to be negatively associated with markers of muscle breakdown in CCI, and the administration of exogenous thyroid hormone was found to be beneficial in reducing hypercatabolic marker levels (24). Growth hormones can promote body growth and metabolism, either directly or indirectly, by stimulating the production of IGF-1. Unlike during the acute phase of sepsis, the secretion of growth hormones is inhibited during CCI and it also leads to low levels of IGF-1 in the circulation (24). Reduced growth hormone and IGF-1 levels are considered to contribute to the development of both muscle atrophy and weakness (25). In addition, the levels of testosterone, a strong anabolic hormone and stimulant of skeletal muscle hypertrophy, were found to be decreased during CCI owing to the inhibition of the hypothalamic-pituitary-gonad axis (26). Long-term low levels of testosterone contribute to the development of hypercatabolism in patients with PICS.

Unlike the changes in the levels of the above hormones, the levels of glucocorticoids were found to increase in most patients with CCI, while adrenocorticotropic hormone (ACTH) levels decreased (27). Elevated circulating glucocorticoid levels are considered to be associated with the hypercatabolic state in patients with CCI despite their potent anti-inflammatory effect (28). Excessive glucocorticoid exposure results in insulin resistance and hyperglycemia in patients with CCI (29), which, consequently, increases the generation of lactate. It also induces muscle wasting by increasing protein breakdown and decreasing protein synthesis (28). For example, the levels of glucagon-like peptide-1 (GLP-1), a catabolic marker produced by intestinal cells, were found to be high from sepsis to CCI (30). Moreover, lipolysis is also upregulated, causing increased blood concentrations of free fatty acids and triglycerides under the release of stress hormones such as (nor)epinephrine and glucagon during sepsis. Both are typical pro-lipolytic hormones, promoting lipolysis by stimulating fat cells directly (10). Hence, endocrine disorders in patients with PICS are responsible for their hypercatabolic status.

### Mitochondrial Dysfunction Changes Energy Metabolism

Mitochondria provide a large proportion of adenosine triphosphate (ATP) to meet cellular energy demands through the electron transport chain (ETC) and oxidative phosphorylation (OXPHOS). However, critically ill patients display significant mitochondrial dysfunction, which is characterized by the reduced expression and activity of ETC complexes, increased ROS generation, and autophagy (31). The expression and activity of ETC complexes I, III, and IV were observed to be significantly reduced in skeletal muscle biopsy specimens of critically ill patients, and those of non-survivors showed a more profound reduction (32, 33). The reduced expression and activity of ETC complexes limit ATP production, consequently leading to decreased energy generation. To meet basic energy demands, the body has to shift metabolic pathways and intensify the catabolism of macronutrients, leading to the hypercatabolic status in PICS.

As a result of mitochondrial dysfunction in sepsis, the levels of lactate and pyruvate in the serum and plasma were found to be increased (34). Pyruvate is unable to generate energy through the OXPHOS but does so through glycolysis. Cells prefer to use glycolysis instead of OXPHOS for generating ATP under aerobic conditions, which is known as the Warburg effect (35). The Warburg effect, also known as aerobic glycolysis, was found to be intensified in septic patients and sepsis survivors (36) due to mitochondrial dysfunction (37). This alteration in cells makes them generate energy rapidly, increases the level of nicotinamide adenine dinucleotide phosphate (NADPH), and affects the generation of ROS (38); however, it also causes pyruvate to build up and lactate levels to rise.

**FIGURE 1:**
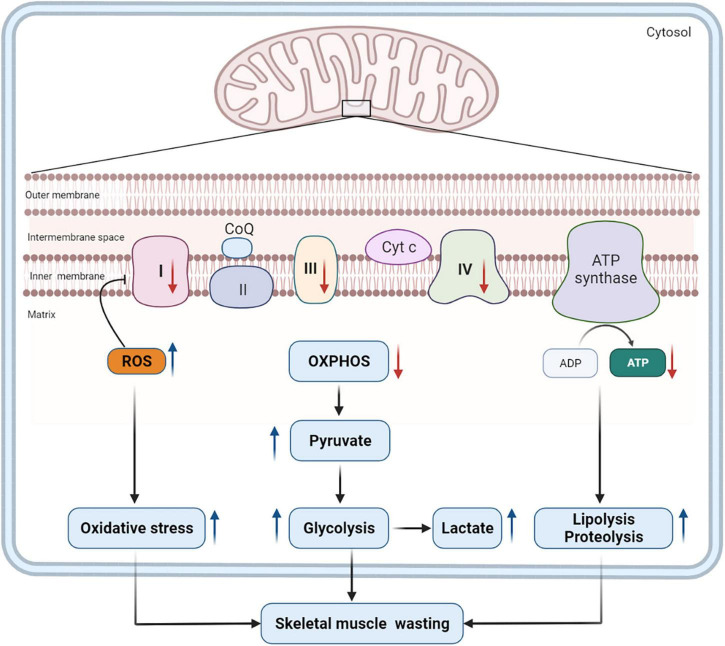
Mitochondrial dysfunction and its impact on catabolism. Mitochondrial dysfunction in PICS decreases the production of ATP by inhibiting the expression and activity of some key enzymes in the ETC (complexes I, III, and IV) (130). Consequently, pyruvate is unable to generate energy through the OXPHOS but does so through glycolysis, and the levels of lactate and pyruvate increase as a result. Mitochondrial dysfunction also contributes to the overproduction of ROS, which, in turn, will inhibit the expression and activity of ETC complexes. Excessive ROS induces oxidative stress, which is harmful to skeletal muscle proteins. To meet tremendous energy demands under the condition of mitochondrial dysfunction, the body has to alter metabolic pathways and intensify the catabolism of macronutrients. These pathophysiological changes will contribute to long-term skeletal muscle wasting in patients with PICS. ROS, reactive oxygen species; CoQ, coenzyme Q; Cyt c, cytochrome c; OXPHOS, oxidative phosphorylation; ATP, adenosine triphosphate; ADP, adenosine diphosphate; ETC, electron transport chain; PICS, persistent inflammation, immunosuppression, and catabolism syndrome.

Mitochondrial dysfunction also decreases the level of antioxidant defense and increases the generation of ROS, which is also affected by the IL-6-mediated gp130/JAK2/STAT3 signaling pathway. The excessive production of ROS is detrimental to skeletal muscles, which contain dense mitochondria and consume much energy. Excessive ROS production impairs mitochondrial ETC proteins directly and causes ETC abnormalities (31), which, in turn, aggravates mitochondrial dysfunction and energy shortage. And oxidative stress induced by mitochondrial dysfunction is harmful to skeletal muscle proteins, causing further muscle wasting (39). Therefore, mitochondrial dysfunction and the consequent oxidative stress may contribute to changes in energy metabolism and skeletal muscle wasting in patients with PICS (Figure 1).

### Gut Dysfunction Limits Nutrient Absorption and Affects Catabolism

Gut dysfunction is common in ICU patients and is related to poor outcomes in critically ill patients. It has been estimated that more than 50% of mechanically ventilated patients experience some degree of gut dysfunction (40). The manifestations of gut dysfunction include delayed gastric emptying, feeding intolerance, impaired small intestinal absorption, and severe diarrhea (41). Chronic critically ill patients who survived initial insults experienced different degrees of gut dysfunction. Slow gastric emptying, a common accompaniment to critical illness, delays the delivery of food to the small intestine and affects digestion and absorption (42). Gut hypoperfusion, which is common in patients with severe sepsis and CCI, may trigger a series of events. Gut hypoperfusion decreases the intestinal blood flow, leading to reduced absorption in the small intestine (42), and it may act as a significant initial event that results in intestinal barrier function compromise. In addition, the promoted gut epithelial apoptosis and inhibited crypt proliferation also contribute to intestinal barrier dysfunction (43), leading to an increase in gut permeability and further hindering nutrient absorption from the small intestine (44). The reduced absorption of nutrients then decreases anabolism. To meet the basic energy demand, there is a consequent increase in catabolism.

The presence, composition, and function of gut microbiota significantly affect the host’s energy metabolism (45). However, the composition and function of gut microbiota were found to be greatly altered in patients with CCI (46). Accumulating evidence has suggested that the disturbance of gut microbiota is closely related to many metabolic disorders, such as insulin resistance, dyslipidemia, skeletal muscle wasting, and malnutrition. For example, short-chain fatty acids (SCFAs), a category of microbiota-derived metabolites, are crucial for glucose homeostasis, lipid metabolism, and skeletal muscle function. They can inhibit intracellular lipolysis, contributing to the accumulation of lipids (47). SCFAs also increase muscle cell insulin sensitivity and glucose metabolism by stimulating the secretion of peptide YY and GLP-1, which affects skeletal muscle cell function (48). However, owing to the disturbance of gut microbiota, the production of SCFAs has been proven to significantly decrease in critically ill patients, leading to insulin resistance, skeletal muscle wasting, and malnutrition (49). In addition, *Escherichia coli* was also reported to play a role in the development of skeletal muscle wasting through the IGF-1/PI3K/Akt/mTOR signaling pathway (50). Thus, alterations of gut microbiota impact the metabolic state in patients with PICS.

**FIGURE 2:**
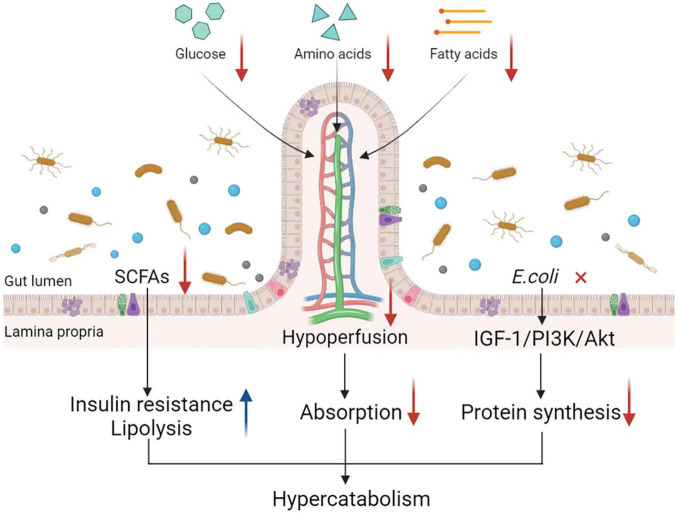
Gut dysfunction and its impact on catabolism. Gut dysfunction contributes to a hypercatabolic state in many ways. Gut hypoperfusion and increased epithelial apoptosis contribute to the intestinal barrier impairment, which is common in critically ill patients, leading to the increase in gut permeability and further hindering nutrient absorption from the small intestine. Alterations in intestinal microbiota such as *Escherichia coli* decrease protein synthesis through the IGF-1/PI3K/Akt signaling pathway. Changes in microbiota-derived metabolites such as SCFAs also lead to the development of hypercatabolism by inducing insulin resistance and lipolysis. Together, these alterations contribute to the hypercatabolic status in patients with PICS. IGF-1, insulin-like growth factor-1; PI3K, phosphoinositide 3-kinase; SCFAs, short-chain fatty acids; PICS, persistent inflammation, immunosuppression, and catabolism syndrome.

In summary, gut dysfunction, manifested as a string of pathophysiological alterations in PICS, limits the digestion and absorption of nutrients and affects the metabolic state of patients with the condition (Figure 2). All these changes imply that the dysfunctional status of the gut may have an important role to play in the development of hypercatabolism in PICS. Gut dysfunction is receiving increasing attention and further studies are needed to understand its exact role in PICS.

### The Ubiquitin-Proteasome and Autophagy-Lysosome Systems

The ubiquitin-proteasome and autophagy-lysosome systems also play important roles in the decomposition of proteins and organelles in skeletal muscles (13). The ubiquitin-proteasome system is the main pathway of protein degradation in muscle cells. In critically ill patients, the expression of forkhead box protein O (FOXO) is upregulated, which can increase the activity of muscle atrophy F-box (MAFbx, also called atrogin-1) and muscle ring finger 1 (MuRF1) (51). MAFbx and MuRF1 are two key E3 (ubiquitin ligating enzyme) ubiquitin ligases, and they play a crucial role in muscle atrophy (52). The expression of MuRF1 can be induced by inflammatory cytokines. The activity of MAFbx and MuRF1 has been observed to be upregulated in muscle atrophy (53). The dysregulation of autophagy-lysosome systems, including excessive autophagy and inadequate autophagy, also contributes to muscle wasting in critically ill patients by impairing myofiber homeostasis. Excessive autophagy leads to the removal of cellular components needed for normal activities, which will limit the function of cells and cause muscle atrophy in the long term (51). And inadequate autophagy fails to remove impaired proteins and leads to the accumulation of dysfunctional cellular components which are toxic to skeletal muscle cells (51).

## Hypercatabolism Is Related to the Progress of Persistent Inflammation, Immunosuppression, and Catabolism Syndrome

In recent years, it has been found that skeletal muscle wasting in patients with PICS is not just a clinical manifestation but a process that is related to the progress of the disease.

### Hypercatabolism Modulates Inflammation

Increased skeletal muscle wasting in patients with PICS releases its pro-inflammatory decomposition products into the circulation and stimulates a sequence of inflammatory responses (1). The insufficient energy supply and muscular injury drive the liberation of damage-associated molecular partners (DAMPs) (54), such as mitochondrial DNA and mitochondrial transcription factor A (55). The released DAMPs bind to pattern-recognizing receptors (PRRs) and are recognized by the host. These receptors include toll-like receptors (TLRs) and nucleotide-binding oligomerization domain-like receptors (NLRs) (56, 57). These DAMPs function as endogenous alarmins (58). When the host recognizes the DAMPs, PRRs will initiate a series of complex downstream signaling events to induce inflammatory responses (56). In addition, hypercatabolism causes malnutrition, and even cachexia, making patients with PICS susceptible to infections. Recurrent infections lead to the invasion of pathogens and induce the release of pathogen-associated molecular patterns (PAMPs), which will also initiate inflammatory responses in the host via PRR signaling pathways (54, 59).

### Hypercatabolism Modulates Immunosuppression

Patients with PICS are in a state of malnutrition, which suppresses the host’s immune response. The imbalance between energy production and utilization contributes to the impairment in immune cell metabolism, affecting immune cell function and triggering a series of pathophysiological alterations (60). Metabolic reprogramming, a characteristic alteration of immune cells in cancer and autoimmune diseases, also acts as a central survival strategy during sepsis. It changes the way immune cells generate ATP, inhibiting mitochondrial respiration and inducing cell cycle arrest (43). It can adjust the priority of energy consumption, limit extra cellular injury and preserve cellular components (55, 61). The Warburg effect, which was first observed in cancer cells, is also a significant part of metabolic reprogramming in immune cells (35). Intensive glycolysis contributes to the increase in lactate levels. In addition, the accumulation of lactate drives immune cells to immunosuppressive phenotype and contributes to immune impairment (62).

In patients with CCI, the high-density lipoprotein (HDL) level was found to be decreased due to the impairment of lecithin-cholesterol acyltransferase activity (63). Decreased HDL levels during critical illness were also associated with suppressed immunity during PICS and regarded as an independent predictor of adverse outcomes and organ failure (64). HDL can regulate immune responses by clearing bacterial toxins and inhibiting monocyte inflammatory responses. When HDL levels drop, these functions are impaired, thereby promoting immunosuppression. Furthermore, persistent inflammation induces inappropriate bone marrow hyperplasia, causing the dramatical expansion of myeloid-derived suppressor cells (MDSCs) in patients with PICS. Over the past several years, MDSC expansion has been found to significantly suppress immunity by inhibiting the proliferation of immune cells (65) and producing anti-inflammatory cytokines such as interleukin-10 (IL-10) (65). A recent study showed that HDL could weaken myelopoiesis and inhibit MDSCs directly (66). Therefore, decreased HDL levels in patients with PICS lead to the freedom of MDSCs from inhibition and cause further immunosuppression.

**FIGURE 3:**
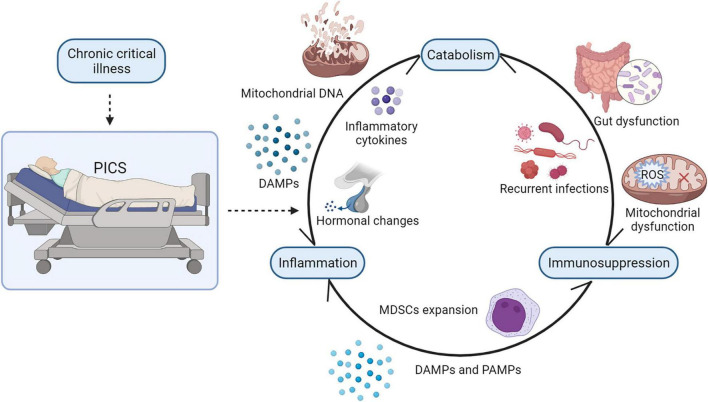
The vicious cycle of PICS. There is a strong interaction between persistent inflammation, immunosuppression, and catabolism. In PICS, ongoing catabolism results in malnutrition and muscle wasting, and its decomposition products such as mitochondrial DNA and other DAMPs may drive persistent low-grade inflammation. Mitochondrial dysfunction and gut dysfunction impair ATP production and, consequently, suppress immunity. Persistent inflammation induces the release of inflammatory cytokines and hormonal changes in patients with PICS, contributing to hypercatabolism. Besides, persistent inflammation also induces inappropriate bone marrow hyperplasia, causing the dramatic expansion of MDSCs in patients with PICS, which will inhibit the proliferation of immune cells. Suppressed immunity causes recurrent infections and inflammatory responses, which consume energy and nutrients. PICS, persistent inflammation, immunosuppression, and catabolism syndrome; MDSCs, myeloid derived suppressor cells; DAMPs, damage-associated molecular patterns; PAMPs, pathogen-associated molecular patterns. SCFAs, short-chain fatty acids; ROS, reactive oxygen species.

In sum, catabolism modulates inflammation and immunosuppression in many ways. In recent years, it has been widely discussed that the three elements of PICS, persistent low-grade inflammation, immunosuppression, and catabolism, may exist in reciprocal causation and form a vicious cycle (1). Once this complicated cycle is initiated, it is hard to reverse (Figure 3). In the future, further studies on how to break the vicious cycle of PICS should be conducted.

## Therapies Focusing on Hypercatabolism

Persistent inflammation, immunosuppression, and catabolism syndrome is an intricate syndrome; thus, a single treatment can hardly interrupt its vicious cycle. A multi-modal therapy is necessary to limit its progression. In addition to eradicating cause and modulating immunity, nutritional support and other therapies to correct hypercatabolism in PICS are also attracting researchers’ attention. Therapies targeted at improving the hypercatabolic status in PICS are summarized in Table 2.

**TABLE 2:** Therapies for hypercatabolism.

Classification	Supplements	Dosage	Main functions	References
**Nutrition**				
	Protein supplements	≥1.3 g/kg/d	Promote nitrogen balance and protein synthesis	(71)
	Arginine	6–9 g/d	Immunologic regulator; Improve insulin sensitivity; Work in coordination with arginine to stimulate the mTOR signaling pathways	(74, 78, 118)
	Leucine	1.2–6 g/d	Promote muscle growth by activating the mTOR signaling pathway	(78, 119)
	Glutamine	0.2–0.4 g/kg/d	Antioxidant; Immunomodulator; Substrate for gluconeogenesis	(120)
	Glucose	≤5 mg/kg/min	Preferential substrate for energy production	(71)
	Lipid	≤1.5 g/kg/d	Energy substrate	(71)
	Omega 3 fatty acids	2 g/d or 0.2 g/kg/d	Immunomodulator; Minimize muscle wasting; Inhibit oxidative injury	(72, 80)
**Anti-inflammatory agents**				
	Anakinra	2.0 mg/kg/h^#^	IL-1 receptor antagonist; Inhibit inflammatory responses	(84)
	Tocilizumab	Unknown	IL-6 receptor inhibitor; Inhibit inflammatory responses	(85)
**Anabolic agents (including hormones)**				
	Propranolol	0.5–3 mg/kg/d	Inhibit lipolysis; Promote protein synthesis; Counteract insulin resistance	(121)
	Oxandrolone	0.1 mg/kg/12 h	Promote protein synthesis	(122, 123)
	Testosterone	200 mg/week	Promote protein synthesis; Reduce protein catabolism and autophagy	(89, 90)
	IGF-1	40 μg/kg/d	Promote muscle growth by activating the mTOR signaling pathway	(124)
**Antioxidants**				
	Mit Q	Unknown	Alleviate oxidative stress in mitochondria	(60)
	Melatonin	50 mg for 5 days^#^	Inhibit mitochondrial structural damage; Alleviate oxidative stress in mitochondria; Improve ATP production	(31, 125)
**Microbiota modulator**				
	Probiotics	(0.8—1.0) × 10^10^ cfu/d *Lactobacillus*; 3.0 × 10^8^ cfu/d *Lactobacillus* and 3.0 × 10^8^ cfu/d *Bifidobacterium*	Protect gut barriers; Rebuild the damaged microbiome; Inhibit bacterial translocation	(126–129)
**Exercise**				
	Early mobilization	15–31 min/d	Improve muscle strength by activating mTOR signaling pathways; Improve mitochondrial function	(107, 111)

*^#^Under preclinical trials.*

*mTOR, mammalian target of rapamycin; IL-1, interleukin-1; IL-6, interleukin-6; IGF-1, insulin-like growth factor-1; Mit Q, mitochondria-targeted ubiquinone; cfu, colony-forming units.*

### Nutritional Support

Nutritional support has become a routine and important intervention for treating critical illnesses. Proper nutritional support has been proven to help improve gut dysfunction and the hypercatabolic state of patients with PICS. Guidelines from the Society of Critical Care Medicine (SCCM, 2016) suggest that early enteral nutrition (EEN, within 48 h after ICU admission) may benefit critically ill patients by effectively improving the nutritional status, alleviating the inflammatory response, impeding bacterial translocation, and improving gut dysfunction (67, 68). However, guideline recommendations for protein intake in ICU patients are divergent. The Protein Summit (2017) recommends protein supplementation in the range of 1.2–2.5 g/kg/d could preserve muscle mass and decrease the mortality rate of patients with CCI (69). Wolfe et al. (70) proposed that higher protein supplementation could suppress endogenous protein catabolism in a dose-dependent manner. The European Society for Clinical Nutrition and Metabolism (ESPEN, 2019) recommends supplying at least 1.3 g/kg of proteins daily for critically ill patients (71). The findings of these studies support the fact that reasonable protein supplementation is a crucial element of care for CCI, and it may improve long-term outcomes and provide benefits to patients with PICS (72).

Compared with the traditional nutritional support formula, an immune-enhancing diet (IED), which can improve both patients’ nutritional status and immune recovery, is more favorable for patients with CCI. IED, which is composed of arginine, glutamine, omega-3 fatty acids, nucleotides, fish oil, and vitamins, has been shown to impede infections, improve adapted immunity, and shorten ICU stay (67). In recent years, IED has become a recommended nutritional intervention for many surgical patients to improve their prognosis (73).

Arginine serves as an immunologic regulator, promoting lymphocyte proliferation and maturation (74). It also produces nitric oxide in enzymatic reactions and dilates blood vessels to enhance the delivery of oxygen and nutrients to the wound (75). In PICS, arginine might be depleted due to the expression of arginase-1 induced by MDSC expansion. Lymphocytes fail to proliferate without arginine, which will contribute to immunosuppression and the occurrence of infections during PICS (74). However, the clinical use of arginine in septic patients and patients with PICS remains controversial because its products may worsen vasodilation and affect hemodynamics (67). Leucine, a branched-chain amino acid, has been demonstrated to decrease muscle catabolism and hypertrophic muscle growth by promoting protein synthesis (76). During PICS, leucine and its product, β-hydroxy-β-methylbutyrate, are likely to decrease or reverse the hypercatabolic status (77). It has been found that leucine can work in coordination with arginine to activate Akt-mTOR signaling pathways, which can promote protein synthesis and inhibit protein breakdown (78, 79). Therefore, arginine and leucine can be supplemented together to benefit patients with PICS. Moreover, glutamine is also likely to benefit patients with PICS because it may act as an antioxidant, immunomodulator, and substrate for gluconeogenesis (72). To sum up, arginine, leucine, and glutamine can be considered to be beneficial for patients with PICS because of their positive effects on metabolism, which need to be studied further.

Omega-3 fatty acids and specialized pro-resolving mediators (SPMs), as adjunctive therapeutic methods, provide potential benefits to patients with PICS. Omega-3 fatty acids were reported to minimize muscle wasting and inhibit oxidative injury by modulating inflammatory responses (80). SPMs are unique derivatives of omega-3 polyunsaturated fatty acids (PUFAs), which can alleviate inflammation and improve a patient’s functional status (81). They also promote tissue regeneration and limit organ injury, which may impede the progression of PICS (82, 83).

### Anti-inflammatory Agents

Exposure to high levels of inflammatory cytokines contributes to the catabolism that occurs during PICS. Hence, anti-inflammatory treatments may help alleviate the hypercatabolic status, and prevent the development of PICS. Current agents used to regulate the inflammatory response during critical illness mainly target IL-1 and IL-6. In a reanalysis of a prior phase III clinical trial, anakinra, an IL-1 receptor antagonist, was shown to have a positive effect on the prognosis of patients with severe sepsis (84). Tocilizumab, a widely studied inhibitor of the IL-6 receptor, blocks the activation of IL-6-mediated signaling pathways, thereby alleviating inflammatory responses in critically ill patients (85). Several candidate therapies targeting other inflammatory cytokines, such as IL-10, interleukin-2 (86), and TNF-α (87), are under investigation. However, the improper use of anti-inflammatory agents may have adverse effects on patients because it may block other relevant signaling pathways mediated by these inflammatory cytokines. Hence, nanomaterial-based therapies for sepsis have been taken seriously in recent years because of their benefits of targeted delivery of anti-inflammatory agents and the reduction in adverse reactions to these drugs (88).

### Anabolic and Anti-catabolic Agents

Anabolic and anti-catabolic agents can alleviate ongoing muscle breakdown and the hypercatabolic state. It has been described above that the decrease in the levels of certain hormones and the declined production of IGF-1 induce the hypercatabolic state in patients with PICS. Hence, testosterone and IGF-1 supplementation may be helpful. Testosterone, an anabolic-androgen steroid, can promote protein synthesis and reduce protein catabolism and autophagy through androgen signaling pathways (89). Testosterone administration has shown its role in improving muscle catabolism in severe burns (90). IGF-1 treatment has also been reported to attenuate catabolism in severely burned patients (91). Thus, testosterone and IGF-1 supplementation are potential therapies for improving skeletal muscle wasting in patients with PICS. Moreover, growth hormones (92) and thyroid hormones (24) were also reported to have positive effects on metabolism; however, the effects of glucocorticoids remain controversial.

In addition to hormonal therapy, other anti-catabolic agents have been used to alleviate the catabolic status. Propranolol, a β-adrenergic receptor blocker, has proven its effect on inhibiting lipolysis, improving the efficiency of protein synthesis, and the restoration of lean body mass in burned patients (93). In addition, propranolol was found to counteract insulin resistance in a dose-dependent manner (94). Oxandrolone, a testosterone analog, can improve the lean body mass and shorten hospital stay in burned individuals (95–97). A combination of rehabilitative exercise training with propranolol and oxandrolone was suggested to improve muscle strength, power, and protein turnover in children recovering from severe burns (98). In summary, anabolic and anti-catabolic agents are promising in attenuating the hypercatabolic status in patients with PICS, and further studies on this subject are necessary.

### Antioxidants

Efforts have also been made to improve mitochondrial dysfunction and oxidative stress in patients with PICS. Antioxidants have been proposed to relieve oxidative stress caused by mitochondrial dysfunction for decades. In recent years, mitochondria-targeted antioxidants that contain ubiquinone or vitamin E have been used to combat the overproduction of ROS caused by mitochondrial dysfunction (60). Among them, mitochondria-targeted ubiquinone (Mit Q) is the most extensively studied. Septic animals treated with Mit Q showed significantly reduced mitochondrial damage, organ dysfunction, and inflammatory responses (99). However, there is a paucity of clinical trials of Mit Q administered in septic patients. Melatonin is another powerful antioxidant that could inhibit mitochondrial structural damage and improve mitochondrial ATP production during sepsis (31). In addition, there are other potential therapies for mitochondrial dysfunction, such as lifestyle intervention, mitochondria membrane stabilization, mitochondrial biogenesis promotion, and mitochondrial transplantation (31, 100). These potential therapies have also shown some efficacy in critically ill animals or patients (31).

### Microbiota Modulator

Therapeutic approaches targeting microbiota modulation have received increasing attention in recent years. The application of microbial modulators, such as probiotics, aims to restore intestinal microbiota balance and intestinal homeostasis in critically ill patients. Microbiota regulators have two main uses; one is to increase the number of beneficial bacteria and the other is to reduce the number of disease-causing bacteria (101). Probiotics, most commonly *Lactobacillus* and *Bifidobacterium*, inhibit the growth of pathogenic bacteria and prevent gut bacteria from migrating to the blood and other organs to rebuild the damaged gut microbiome and restore normal gut function (102). Some probiotics such as *Lactobacillus* and *Saccharomyces boulardii* have also been reported to promote the secretion of immunoglobulins in the gut, thereby enhancing the immune function of the gut and reducing the harmful effects of toxins and pathogenic bacteria (103). In addition, the nutrition support mentioned above and fecal microbiota transplantation (104) are also important means of promoting the recovery of gut function in patients with PICS. However, these therapies may cause side effects such as diarrhea, constipation, and infections when bringing benefits to patients (104). Hence, more experimental and clinical studies are needed to improve their safety and efficacy in the future.

### Early Mobilization and Other Therapies

Not only hypercatabolism but also physical immobilization contributes to rapid skeletal muscle wasting and impaired functional status in ICU patients. Early mobilization, a promising intervention in the ICU, has been proven to be beneficial to critically ill patients in many studies (105, 106). Early mobilization and exercise promote muscle fiber contraction, which generates mechanical signals to stimulate the activation of the mTOR signaling pathway (107). The activated mTOR signaling pathway has a positive influence on muscle cell hypertrophy and muscle growth. It may improve muscle strength, reduce activity limitations, and shorten ICU stay (108–111). In addition, early physical mobilization might counteract sepsis-induced catabolism (112). A recent randomized controlled trial supposed that early mobilization during the first week of septic shock preserves skeletal muscle mass by limiting the excessive activation of autophagy instead of inhibiting autophagy (113). Therefore, early mobilization has the priority of use in the hospital course of patients with PICS.

**FIGURE 4:**
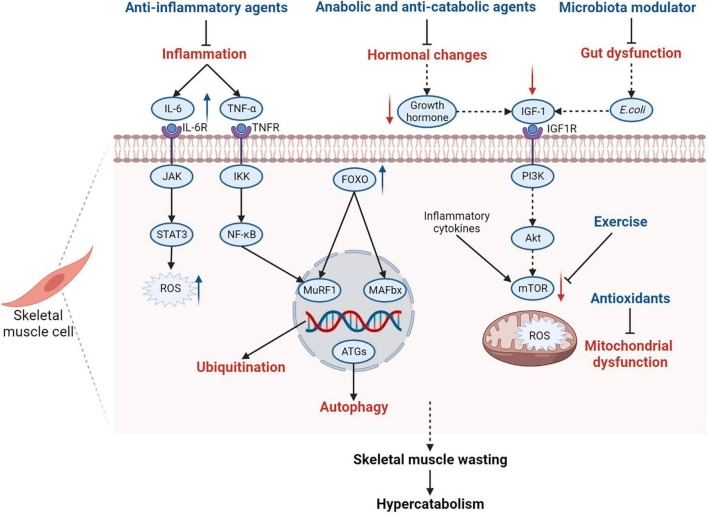
Summary of pathophysiological mechanisms of skeletal muscle wasting in PICS and related therapies. Many pathophysiological changes in PICS affect the function of skeletal muscle cells and contribute to skeletal muscle wasting in patients with PICS. Inflammatory cytokines, such as IL-6 and TNF-α, induce protein degradation by activating the gp130/JAK/STAT3 and IKK/IKB/NF-κB signaling pathways, contributing to the production of ROS in muscle cells. Elevated inflammatory cytokines also inhibit the mTOR-mediated signaling pathway to decrease protein synthesis. Some hormones such as growth hormones are inhibited during PICS, which will cause decreased IGF-1 levels and suppress the IGF-1/PI3K/Akt/mTOR signaling pathway. Alterations in *Escherichia coli*, as a part of gut dysfunction, also lead to the suppression of the IGF-1/PI3K/Akt/mTOR signaling pathway by inhibiting IGF-1 production. Hence, exercise and IGF-1 supplementation are encouraged for patients with PICS to activate the mTOR signaling pathway and promote protein synthesis. In addition, the expression of FOXO is upregulated in skeletal muscle cells, which can increase the activity of MAFbx and MuRF1, activating the ubiquitin-proteasome system as a result. The autophagy-lysosome system is also activated and the levels of autophagy-related genes are upregulated. Mitochondrial dysfunction contributes to the increase in ROS, which induces skeletal muscle wasting and hypercatabolism in PICS. Therefore, anti-inflammatory agents, anabolic and anti-catabolic agents, microbiota modulators, and antioxidants are recommended for patients with PICS. IL-6, interleukin 6; IL-6R, interleukin 6 receptor; JAK, Janus kinase; STAT3, signal transducer and activator of transcription 3; ROS, reactive oxygen species; TNF-α, tumor necrosis factor α; TNFR, tumor necrosis factor receptor; IKK, IκB kinase complex; NF-κB, nuclear factor-kappa B; FOXO, forkhead box protein O; MuRF1, muscle ring finger 1; MAFbx, muscle atrophy F-box; ATGs, autophagy-related genes; IGF-1, insulin-like growth factor-1; IGF1R, insulin-like growth factor-1 receptor; PI3K, phosphoinositide 3-kinase; mTOR, mammalian target of rapamycin; gp130, glycoprotein 130; PICS, persistent inflammation, immunosuppression, and catabolism syndrome.

The ubiquitin-proteasome and autophagy-lysosome systems contribute to skeletal muscle wasting in patients with PICS. In recent years, therapies targeting these two systems have been emerging. Ubiquitin-proteasome system inhibitors such as bortezomib and MuRF1 inhibitors are useful in preventing muscle atrophy (114). Autophagy modulators and other therapies also have been studied to protect skeletal muscles from atrophy (19). These therapies are potentially beneficial to patients with PICS. However, the exact underlying mechanisms and therapeutic schedules need to be further investigated. Therapies targeting the pathophysiological mechanisms of skeletal muscle wasting are shown in Figure 4.

## Recommendations to Avoid Persistent Inflammation, Immunosuppression, and Catabolism Syndrome

Driven by the continuous exposure to DAMPs and PAMPs, patients with PICS are trapped in a vicious cycle of inflammation, immunosuppression, and hypercatabolism, which leads to poor outcomes. Hence, early intervention and interruption are encouraged to prevent the occurrence of PICS. Initial insults such as sepsis, severe burns, and severe trauma may develop into PICS at a later stage. Therefore, aggressive treatment of these initial insults is important in preventing the development of PICS. Early removal of the etiology, proper nutritional supplementation, and early exercise support are necessary. These methods help critically ill patients better fight the disease and avoid transitioning to its chronic stage. However, arginine supplementation is not recommended in the early stages because it may cause hemodynamic instability, which can be detrimental to the patient (67). In addition, patients suffering initial insults may have underlying mitochondrial dysfunction and gut dysfunction; however, these changes may be underappreciated. Therefore, improving mitochondrial dysfunction and gut dysfunction at the initial stage of the condition is also a potential means of preventing the occurrence of PICS. In a nutshell, an early and multipronged therapeutic schedule is required to avoid PICS.

## Conclusion

Persistent inflammation, immunosuppression, and catabolism syndrome, an important phenotype of CCI, has become an arduous problem in the ICU since it was proposed. In this review, we focused on hypercatabolism, which is less mentioned in PICS, and proposed that inflammation, hormonal changes, mitochondrial dysfunction, and gut dysfunction could be important underlying mechanisms. Hypercatabolism is involved in the progress of persistent inflammation and immunosuppression in PICS. Hence, therapies for preventing and improving the hypercatabolic status in patients with PICS are vital. A multi-modal therapy that includes nutritional support, anti-inflammatory agents, anabolic and anti-catabolic agents, antioxidants, microbiota modulators, and early mobilization is suggested to benefit patients with PICS.

## Outlook and Future Directions

The existing difference in diagnostic criteria for PICS reflects the complexity of its pathophysiological changes and difficulty in its treatment. The detection of biomarkers of inflammation, immunosuppression, and metabolism, such as GLP-1, may help clinicians identify pathophysiological changes in patients and prevent the development of PICS. However, it needs further research. Based on the influence of gut microbes on body metabolism, studying this process can provide more valuable suggestions for treating PICS. Microbiota modulators have been suggested as a way to improve gut dysfunction, but the long-term effects of probiotics and their corresponding metabolites on patients with PICS are needed to be fully understood. Some therapies aimed at improving inflammatory response and mitochondrial dysfunction such as anakinra and melatonin are currently in phase III clinical trials. Additionally, studies on the IL-6 inhibitor tocilizumab, ubiquitin-proteasome system inhibitor bortezomib, and mitochondria-targeted antioxidant Mit Q are still in their infancy. However, these limitations do not deter the fact that therapies focusing on catabolism are promising in challenging PICS. In future experiments, based on existing studies, more evidence-based studies on metabolic characteristics are needed to establish effective and standardized interventions for PICS, so as to improve long-term prognosis.

## Author Contributions

JLZ, WL, and JZ conceived and designed the manuscript together. JLZ and JZ wrote and revised the manuscript. WL and CM checked and revised the manuscript. JZ checked and approved the manuscript finally. All authors read and approved the submitted version.

## Conflict of Interest

The authors declare that the research was conducted in the absence of any commercial or financial relationships that could be construed as a potential conflict of interest.

## Publisher’s Note

All claims expressed in this article are solely those of the authors and do not necessarily represent those of their affiliated organizations, or those of the publisher, the editors and the reviewers. Any product that may be evaluated in this article, or claim that may be made by its manufacturer, is not guaranteed or endorsed by the publisher.
